# P-1911. Covid-19 Associated Mucor-Aspergillosis Incidence, Management and Outcome analysis

**DOI:** 10.1093/ofid/ofae631.2072

**Published:** 2025-01-29

**Authors:** Venkat R Kola

**Affiliations:** Yashoda hospitals, Hyderabad, Telangana, India

## Abstract

**Background:**

The clinical course of COVID-19 has been complicated by secondary infections, including bacterial and fungal infections. The rapid rise in the incidence of invasive mucormycosis as well as mixed mucormycosis and aspergillosis in these patients is very much concerning. COVID19-associated Mucor-Aspergillosis was detected in significant numbers during the second wave of the COVID-19 pandemic in India, with several predisposing factors indicated in its pathogenesis. This study aimed to evaluate the epidemiology, predisposing factor, cumulative mortality and factors affecting outcomes among the coronavirus disease COVID-19-associated mucor-aspergillosis(CAMA).

Histopathology image showing aspergillosis and mucormycosis

**Figure:**
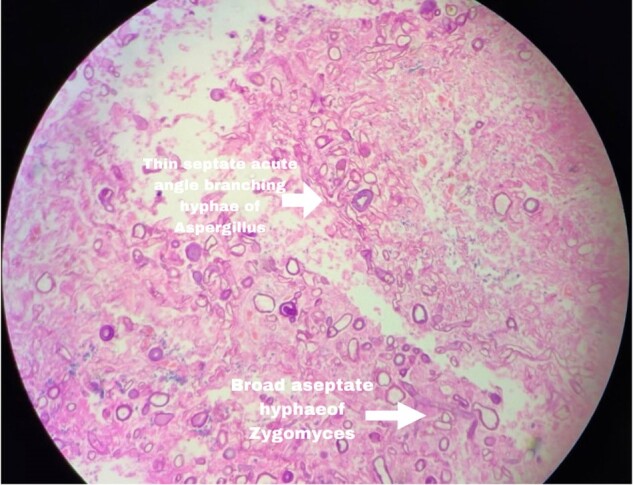

Histopathology of CAMA

**Methods:**

A multicenter ambispective study across three tertiary health care centers in Southern part of India was conducted during April-October 2021and the patients were followed up for a period of 30 months till April 2024.

Surgical Debridement specimen (Maxillectomy) of CAMA

**Figure:**
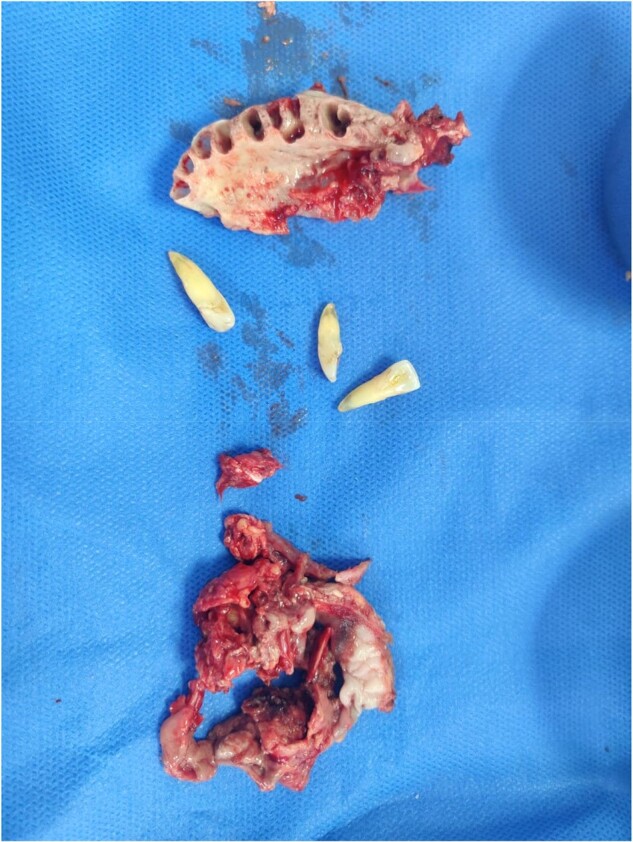

Surgical Debridement specimen (Maxillectomy) of CAMA

**Results:**

Among the 56 cases of CAMA, mucor-aspergillosis affecting the nasal sinuses was the commonest, affecting 29(51%) of the patients, orbital extension seen in 15(26%), pulmonary (n=8, 14%), gastrointestinal (n=2, 3%), isolated cerebral (n=1) and disseminated mucor-aspergillosis in 1 patient. Diabetes mellitus, high-dose systemic steroids were the most common underlying disease among CAMA patients.

The CAMA associated case-fatality at 6 weeks was 14%, cerebral or GI or disseminated mucor-aspergillosis had 9 times higher risk of death compared to other locations. Extensive surgical debridement along with sequential antifungal drug treatment improved the survival in mucor-aspergillosis patients.

**Conclusion:**

The incidence of mucor-aspergillosis increased immensely during the second wave of COVID in India, and hence clinicians should be aware and have up-to-date knowledge of the predisposing factor, clinical signs and symptoms, diagnostic modalities and treatment strategies of the various types of CAMA. Also, the treating physicians should remember the predisposing factor involved in the development of CAMA, the improper use of glucocorticoid, and should manage COVID cases accordingly with the appropriate drugs, thus preventing increase in CAMA cases. Multi-disciplinary approach and timely surgical and medical management can be much helpful in achieving a successful outcome.

**Disclosures:**

All Authors: No reported disclosures

